# On the nature of high-amplitude propagating pressure waves in the human colon

**DOI:** 10.1152/ajpgi.00386.2019

**Published:** 2020-02-18

**Authors:** Natalija Milkova, Sean P. Parsons, Elyanne Ratcliffe, Jan D. Huizinga, Ji-Hong Chen

**Affiliations:** McMaster University, Department of Medicine, Division of Gastroenterology, Farncombe Family Digestive Health Research Institute, Hamilton, Ontario, Canada

**Keywords:** bisacodyl, colonic motility, high-amplitude propagating pressure waves, high-resolution colonic manometry, human colon

## Abstract

Characterization of high-amplitude propagating pressure waves (HAPWs or HAPCs) plays a key role in diagnosis of colon dysmotility using any type of colonic manometry. With the introduction of high-resolution manometry, more insight is gained into this most prominent propulsive motor pattern. Here, we use a water-perfused catheter with 84 sensors with intervals between measuring points of 1 cm throughout the colon, for 6–8 h, in 19 healthy subjects. The catheter contained a balloon to evoke distention. We explored as stimuli a meal, balloon distention, oral prucalopride, and bisacodyl injection, with a goal to optimally evoke HAPWs. We developed a quantitative measure of HAPW activity, the “HAPW Index.” Our protocol elicited 290 HAPWs. 21% of HAPWs were confined to the proximal colon with an average amplitude of 75.3 ± 3.3 mmHg and an average HAPW Index of 440 ± 58 mmHg·m·s. 29% of HAPWs started in the proximal colon and ended in the transverse or descending colon, with an average amplitude of 87.9 ± 3.1 mmHg and an average HAPW Index of 3,344 ± 356 mmHg·m·s. Forty-nine percent of HAPWs started and ended in the transverse or descending colon with an average amplitude of 109.3 ± 3.3 mmHg and an average HAPW Index of 2,071 ± 195 mmHg·m·s. HAPWs with and without simultaneous pressure waves (SPWs) initiated the colo-anal reflex, often abolishing 100% of anal sphincter pressure. Rectal bisacodyl and proximal balloon distention were the most optimal stimuli to evoke HAPWs. These measures now allow for a confident diagnosis of abnormal motility in patients with colonic motor dysfunction.

**NEW & NOTEWORTHY** High-amplitude propagating pressure waves (HAPWs) were characterized using 84 sensors throughout the entire colon in healthy subjects, taking note of site of origin, site of termination, amplitude, and velocity, and to identify optimal stimuli to evoke HAPWs. Three categories of HAPWs were identified, including the associated colo-anal reflex. Proximal balloon distention and rectal bisacodyl were recognized as reliable stimuli for evoking HAPWs, and a HAPW Index was devised to quantify this essential colonic motor pattern.

## Graphical abstract

**Figure:**
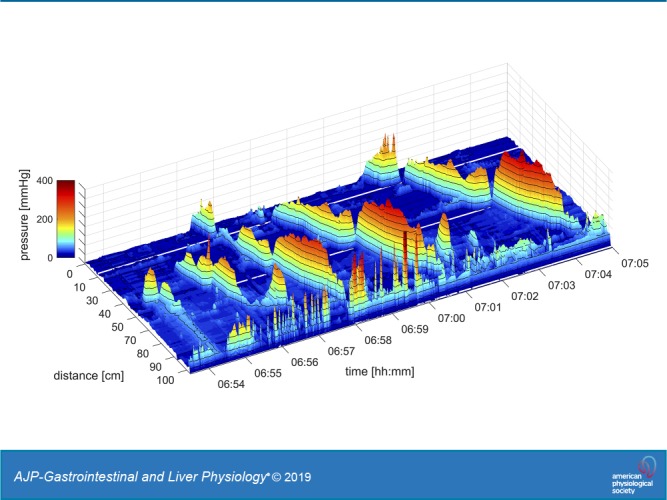

## INTRODUCTION

Chronic colonic motility disorders are treated or undergo surgical intervention, most often without proper diagnosis of motor dysfunction, yet all consensus reports indicate that colonic manometry is essential for diagnosis of colon motor dysfunction (11, 19, 43, 47). Colonic manometry is considered to be of uncertain usefulness because of our limited knowledge of normal colon motor patterns and normal reaction to stimuli. Diagnosis of esophageal dysfunction has changed due to high-resolution manometry, from measurements of isolated points along the esophagus due to a low number of sensors, to a detailed characterization of esophageal motility. This, among other improvements, led to increased sensitivity to detect achalasia, and it allowed for subclassification of achalasia leading to improved guidelines for treatment (27, 57). The equivalent of esophageal peristalsis in the colon is the high-amplitude propagating pressure wave (HAPW) (22, 44), also known as high- amplitude propagating contraction (HAPC) (1, 6), or high-amplitude propagating sequence (HAPS) (19, 23). Guidelines for colonic manometry indicate that the most important feature that should be achieved is the ability to conclude that a patient’s motor function is normal (11). However, we do not yet have criteria to confidently identify normal HAPWs, and no consensus exists as to which protocol to use to elicit HAPWs for diagnostic purposes. Also, an adequate healthy control data set is essential for interpreting an abnormal test (11), and such a data set is not yet available. High-resolution colonic manometry (HRCM) may achieve this, and the present study provides an important advance toward this goal. Previous studies have demonstrated the relevance of appreciating the regional distribution of propagating waves in the colon. It was found that in the early predefecatory phase, the origin of HAPWs shifts distally (1). This coordinated spatiotemporal pattern has been suggested to play an important role in the shifting of colonic content in the rectal direction to prepare for defecation, as most individual HAPWs do not span the entire colon (9). The innervation of the colon also shows regional differences (9, 58), and functional differences related to transit and storage are well documented (5, 7, 41). There have also been indications in the pediatric literature that HAPWs are not normal unless they span the entire colon (47), which makes it important to study regional HAPWs. Therefore, the first objective of this study was to characterize HAPWs in healthy subjects using 84 sensors throughout the colon based on the site of origin and site of termination and quantify their features, so as to assess in future studies potential regional dysfunction in patients. HAPWs generally occur between 4 and 10 times per 24 h in the unprepared colon (23, 35, 45). In a short manometric study, they may not happen without a stimulus and are usually evoked by various stimuli, including a meal and proximal bisacodyl. However, in healthy subjects, a meal may not evoke HAPWs, and, rarely, bisacodyl may not either (6). Hence the second objective was to identify optimal stimuli that will reliably evoke HAPWs in healthy subjects. A third objective was to develop a quantitative assessment of normal HAPW activity.

## METHODS

### Study Subjects

Nineteen healthy subjects (aged 21–54 yr; 9 women, 10 men) were recruited by local advertising. All participants gave written informed consent, and all procedures were approved by the Hamilton Integrated Research Ethics Board. Exclusion criteria included abdominal surgery, hepatic, kidney, or cardiac diseases, connective tissue disorders, central nervous system disorders, thyroid diseases, prostate diseases, or any malignancies. All subjects reported normal stool consistency (Bristol type IV) and normal bowel frequency: between one bowel movement every 3 days and three bowel movements per day. None had defecation difficulties, and none were taking medication that might influence bowel movements. Before the start of the study, subjects were briefed on details of the study, and informed that there may be discomfort due to water expulsion by motor activity.

### High-Resolution Colonic Manometry

High-resolution colonic manometry (HRCM) was performed on a custom-made platform [Medical Measurement Systems (MMS); Laborie, Toronto, Canada]. A two-balloon catheter was used in the first 13 subjects, which included balloons between sensors 10 and 11 and 40 and 41. All other volunteers had one balloon between sensors 10 and 11. All balloons were 10 cm in length. The water perfusion rate was 0.1 ml/min via each sensor, resulting in a maximum total of 0.5 L/h when all pressure sensors were inside the colon; the perfusion pressure was 1,000 mbar (100 kPa). Each manometry study lasted 6–8 h, resulting in 3–4 L of water being delivered into the colon. Water was expelled via propulsive motor patterns and through a drainage tube (3.3 mm × 91 cm; Salem Sump, Covidien) placed in the rectum that diverted 1–2 L of water. Water will also have been absorbed. The intraluminal pressure between motor patterns did not change for the duration of the 6–8-h manometry session, measured by baseline pressure readings at the start and end of the procedures; thus, the inflow of water did not cause passive tonic pressure changes that might have evoked motor activity.

The catheter was inserted with minimal sedation (fentanyl 50–100 µg iv and midazolam 2–5 mg iv) with the assistance of a colonoscope after a bowel-cleaning procedure using an inert osmotic laxative (PEG-Lyte; Pendopharm, Quebec, ON, Canada) but no stimulant laxatives, such as bisacodyl. Three liters of PEG (70 g/L) were taken between 4 and 6 PM the day before the procedure, with more water consumed as needed so that all solids would be removed. The next morning, 1 L was taken at 4 AM. The tip of the catheter was clipped to the mucosa via a fish line tied to the tip of the catheter, a few centimeters distal to the cecum. The anal sphincter was recorded across 2–4 sensors; therefore, although catheter displacement was rare, movement of the catheter could be detected through a shift in those sensors. The catheter was made of 100% silicone; after use, a hospital-approved cleaning procedure was executed, including sterilization with an autoclave. All subjects were in the supine position during the entire recording, with the exception of the intake of the meal when they were seated upright at a 45° angle. The subjects were instructed to report all events, such as gas or liquid expulsion, cramping, and nausea. The subjects were asked to refrain from preventing or promoting gas or liquid expulsion by increasing abdominal pressure or contracting the external anal sphincter should an urge arise. All body movements, such as changing body position, talking, coughing, laughing, and urination were noted immediately into the data acquisition files to remove pressure artifacts.

### Protocol

A 90-min recording of baseline activity was started 30 min after the colonoscope was withdrawn. The response to a 5-min balloon distension at the proximal colon was investigated. The balloon was initially inflated until the first sensation was reported. This was followed by incremental increases in balloon volume by 60 mL until the maximum tolerated volume was achieved, which was between 250 and 400 ml air. In each of these periods, the volume was sustained for a short period (between 2 and 3 min). The maximum tolerated volume was considered to be when discomfort reached 6–7 on a 10-point scale, as reported by the subject. After the 5-min distention, the balloon was deflated. Analysis of the response to balloon distention was performed on the 5-min period of sustained distention, as well as a 15-min period after deflation. Next, a meal was given to induce the gastrocolonic reflex (500 g of organic vanilla yogurt fortified with organic milk fat (Mapleton Organics, Moorefield, ON, Canada), to reach 800–1,000 kcal). Its effect was observed for 90 min. Next, 4 mg of prucalopride was administered orally, and its effect was observed for 90 min; prucalopride was given to study a possible early effect due to stimulation of the gastric enterochromaffin cells (15, 25), not to study effects of prucalopride after it is absorbed in the bloodstream. Following prucalopride, a 10-mg bisacodyl suspension (Dulcolax; Boehringer Ingelheim, Sanofi Canada, Quebec) was injected in the rectum via a syringe, and its effect was studied for 30 min. The bisacodyl suspension was made in saline by crushing four tablets, 5 mg each, with a pestle and mortar for 5 min. Since it was not possible to keep patients for multiple days and perform separate interventions, all of the stimuli were administered within a 6–8-h time span; therefore, some of the observed effects such as those of prucalopride and bisacodyl may have acted additively. At the end of the study, an X-ray was taken using a portable X-ray machine that was brought into to the study room. Before the X-ray, one or both balloons were slightly inflated so as to make them visible during X-ray and were used along with the catheter clip and metal pieces to visualize the placement of the catheter along the colon. Metal pieces were incorporated in the catheter at the tip and at both sides of the balloon(s) to help identify the position of the catheter.

### Analysis

The manometric recording was first inspected visually to identify all motor patterns and artifacts. Artifacts due to cough, position change, or straining were removed from analysis. An HAPW was identified as a motor pattern that propagated slower than 2.5 cm/s, has an average pressure of more than 20 mmHg (based on the MMS topographical map pressure scale), and was not part of a cyclic motor pattern (24, 38). HAPWs occurred with or without a subsequent simultaneous pressure wave (SPW) (14). To analyze all of the motor patterns, an Event Series plug-in was used in ImageJ, which converts the data from the manometry scan into a spatiotemporal plot and allowed us to use the tools provided by ImageJ to measure various parameters. To measure HAPW amplitude, the freehand tool was used to outline the general area around the pressure wave. A 20-mmHg isobaric contour line was then set using a Contourer plug-in, to measure the average amplitude of each individual HAPW within this isobar. To measure its velocity, the line tool was used to draw a line from the beginning of the pressure wave to the most distal end. From the line tool, we obtained the length (over how many centimeters of the colon the wave progressed) and its duration (the time difference between the start and end) of the pressure wave. From these data, we also calculated the velocity using length/duration.

Pressure waves were categorized according to points of origin and cessation in the colon, as well as the intervention during which they occurred. The exact positioning of the catheter within the colon was determined on the basis of the X-ray taken at the end of the study. HAPWs were paired with their associated percentage anal sphincter relaxation (the colo-anal reflex) which was measured using ImageJ; its rectangular selection tool was used to obtain the mean amplitude of the relaxation, as well as the anal sphincter amplitude 3 min before the relaxation occurred (reference amplitude). To measure the mean amplitude of the relaxation pressure, the plot profile option in ImageJ was used to narrow the selection to only encompass the lowest area of pressure associated with the HAPW, and this area was taken as the area of relaxation. To measure the reference pressure, the area of the anal sphincter 3 min before the relaxation was taken. If HAPWs occurred at a higher frequency, or if there was another motor pattern occurring just before the HAPW, the resting pressure that was available between the two consecutive relaxations was taken as the reference. The percent relaxation was calculated using the formula:

$$100-\left[\left(\frac{\text{relaxation amplitude}}{\text{reference amplitude}}\right)\times 100\right].$$

Twenty percent anal sphincter relaxation was considered clinically relevant as per anorectal manometry guidelines (42).

### Statistical Analysis

Data were expressed as means ± SE, with *N* as the number of subjects and *n* as the number of HAPWs. Normal values for each intervention and category were determined using the 95th percentile. GraphPad Prism 8 was used for statistical analysis. The Brown-Forsythe test was used to test for significant differences between the variances of the groups. If the test did show a significant difference in variances, Welch’s one-way ANOVA was used with Games-Howell post hoc test for multiple comparisons. An unpaired *t* test with or without Welch’s correction was used to compare the percentage of anal sphincter relaxation between independent and HAPW-associated relaxations, as well as differences between HAPWs with and without SPW within each category depending on whether there was a significant difference in variances. Variances for the *t* test were compared using the *F* test.

### Generation of Symbol Maps

The present study devised symbol maps to show the occurrence of HAPWs on a time scale and to illustrate the variability in responses of healthy subjects. Isolated SPWs were also included as characterized in a previous study (13).

### HAPW Index Calculation

In esophageal high-resolution manometry, topographical maps are used to calculate an index of motility called the distal contractile integral (DCI) (17, 26, 37). The DCI assesses the vigor with which a contraction occurs, and it is measured by multiplication of the amplitude, length, and duration of the pressure wave (mmHg·cm·s) (28, 32, 36). It is used in combination with other factors to ascertain whether a patient is suffering from a certain deglutitive disorder (36). The present study set out to determine a similar index to be used for the assessment of the HAPWs, the HAPW Index. It was found that HAPWs of highest likelihood to be propulsive are those of high amplitude and a longer duration (18). The HAPW Index was calculated as the average amplitude within the 20-mmHg isobar times the duration times the length of the HAPW. In the esophagus, data are expressed as millimeters of mercury times centimeter times second. Given that the HAPW Index in the colon was ~100 times higher than the esophageal DCI, we expressed the Index as millimeters of mercury times meter times second, so as to avoid very large unwieldy numbers.

## RESULTS

**Fig. 1.**
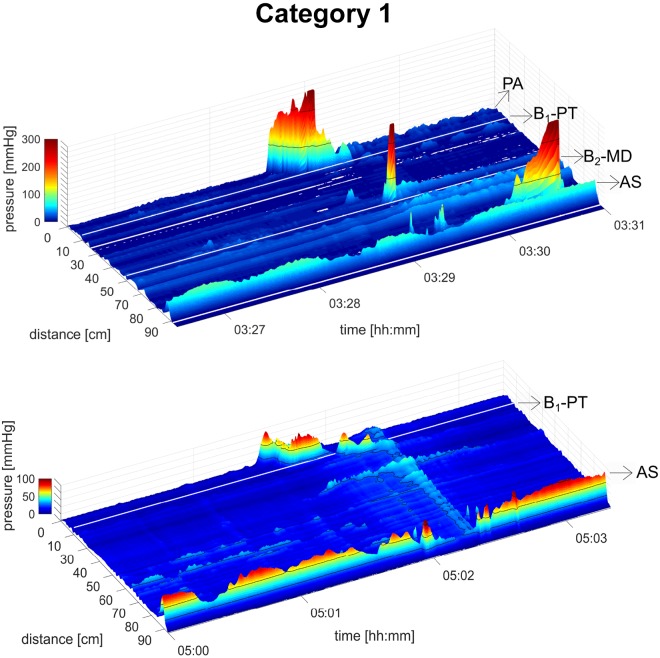
*Category 1*: proximal high-amplitude propagating pressure waves (HAPWs). HAPWs originate in the ascending colon and also terminate within it. They may terminate fully (*A*), or transform into a simultaneous pressure wave (SPW; *B*). *A* was observed during a meal, and *B* was observed during rectal bisacodyl injection. White line represents 10-cm balloon. AS, anal sphincter; MD, mid-descending; PA, proximal ascending; PT, proximal transverse. B_1_, balloon 1; B_2_, balloon 2.

**Fig. 2.**
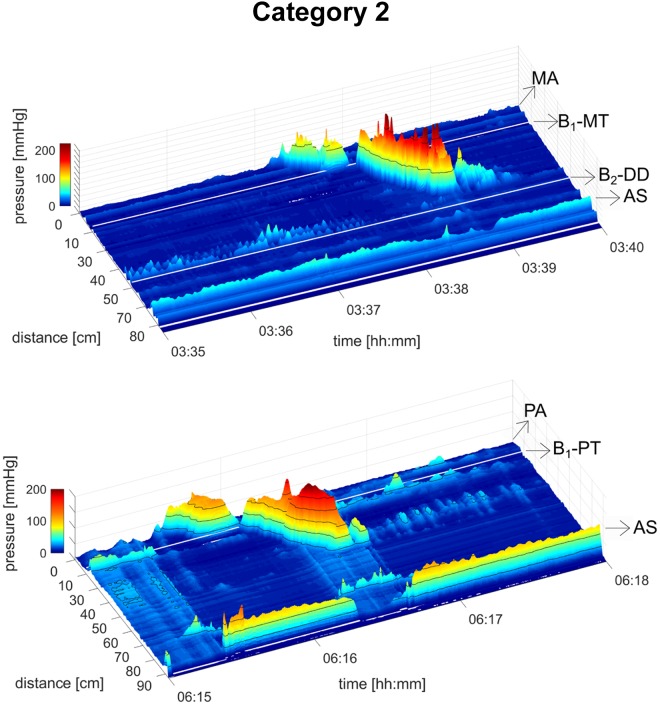
*Category 2*: proximal continuing high-amplitude propagating pressure waves (HAPWs). HAPWs originated in the ascending colon and may terminate in the transverse, descending, sigmoid colon, or rectum either fully (*A*) or transform into a simultaneous pressure wave (SPW; *B*). *A* was observed during meal, and *B* was observed during rectal bisacodyl. White line represents 10-cm balloon. AS, anal sphincter; DD, distal descending; MA, mid-ascending; PA, proximal ascending; MT, midtransverse. B_1_, balloon 1; B_2_, balloon 2.

**Fig. 3.**
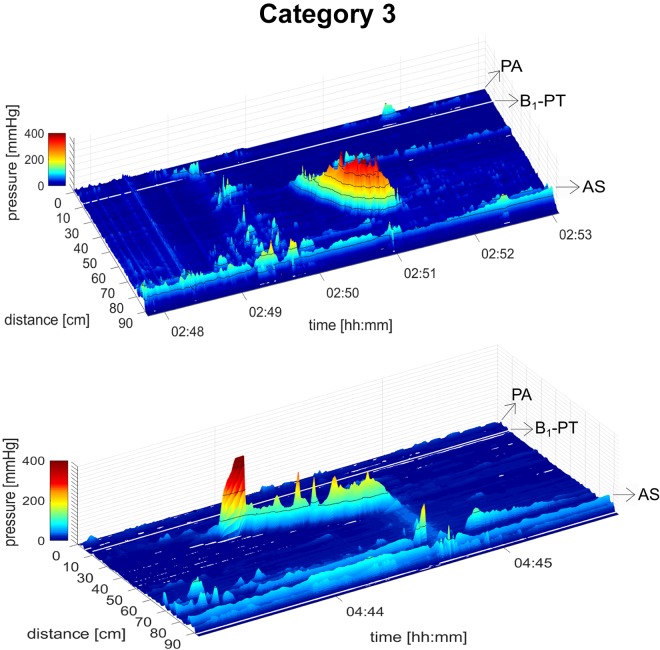
*Category 3*: transverse/descending high-amplitude propagating pressure waves (HAPWs). HAPWs originate in the transverse or descending colon and may terminate fully (*A*) or transform into a simultaneous pressure wave (SPW; *B*). *A* was observed during meal, and *B* was observed during oral prucalopride. White line represents 10-cm balloon. AS, anal sphincter; PA, proximal ascending; PT, proximal transverse. B_1_, balloon 1; B_2_, balloon 2.

A total of 19 healthy subjects underwent HRCM, which generated 290 HAPWs (Figs. 1–3). HAPWs, independent of location or type, had an average amplitude higher than 50 mmHg, and a velocity between 0.2 cm/s and 2.2 cm/s. HAPWs were associated with an average anal sphincter relaxation of 66% (range 61–100%), from an average resting anal sphincter pressure of 52.8 ± 2.0 mmHg (range: 48.9–56.8 mmHg) measured in the 3-min period before the HAPW. All HAPWs propagated in antegrade direction.

### HAPW Categories

The HAPWs were classified into three different categories based on their origin and termination in the colon, starting with activities that were initiated in the proximal colon.

#### Category 1. Proximal HAPWs: HAPWs originating in the ascending colon that did not propagate beyond it (21%; N = 12 subjects, n = 62 HAPWs).

Examples are shown in Fig. 1. The average amplitude of the HAPWs in this category was 75.3 ± 3.3 mmHg. The normal range based on the 95th percentile was 46.5–145.2 mmHg. The mean velocity was 0.88 ± 0.11 cm/s with a range of 0.32–2.2 cm/s. The mean HAPW Index was 440 ± 58 mmHg·m·s, and its range was 87–1540 mmHg·m·s. The average anal sphincter relaxation for this group was 47.5 ± 3.1%. 89% of the HAPWs in this category were associated with relaxation of the anal sphincter of >20%. 64% of the HAPWs in this category transformed into SPWs; 88% of these were associated with anal sphincter relaxation. In this category, there were no significant differences in amplitude, velocity, or Index between HAPWs with or without SPWs.

#### Category 2. Proximal continuing HAPWs: HAPWs originating in the ascending colon and terminating in the transverse, descending, or sigmoid (29%; N subjects = 13, n = 85 HAPWs).

HAPWs originating in the proximal colon and terminating beyond it were the second most prominent category (Fig. 2). Their mean amplitude was 87.9 ± 3.1 mmHg with a normal range between 52.5 and 141.9 mmHg. The mean velocity of this category was 0.79 ± 0.05 cm/s, range: 0.29–1.50 cm/s. Their mean Index was 3,344 ± 356 mmHg·m·s, range: 368–12,189 mmHg·m·s. 92% of the HAPWs in this category were associated with significant anal sphincter relaxation of more than 20% from resting pressure. 68% of the HAPWs in this category terminated in the transverse or descending colon by transforming into SPWs. 97% of the HAPWs without SPWs were associated with anal sphincter relaxation. In this category, HAPWs without SPWs had significantly higher amplitude (*P* < 0.0001) and HAPW Index (*P* < 0.0001). HAPWs with SPWs had a significantly higher velocity (*P* = 0.0062).

#### Category 3. Transverse and descending HAPWs: HAPWs originating in the transverse or descending colon (49%; N = 18 subjects, n = 143 HAPWs).

This category of HAPWs was the most prominent (Fig. 3). Their mean amplitude was 109.3 ± 3.3 mmHg, and their range was 48.0–183.5 mmHg. The mean velocity of this category was 0.60 ± 0.03 cm/s, and the range was 0.22–1.15 cm/s. The average HAPW Index was 2,071 ± 195 mmHg·m·s and the range was 155–7,492 mmHg·m·s. 95% of HAPWs in this category were associated with relaxation of the anal sphincter of more than 20% from resting pressure. 39% of the HAPWs in this category transformed into SPWs in the descending colon. 96% of HAPWs with SPWs in this category were associated with anal sphincter relaxation or with a contraction. In this category, HAPWs without SPWs had significantly higher amplitude (*P* < 0.0001) and index (*P* < 0.0001); however, velocity was not significantly different.

### Spontaneous Relaxations of the Anal Sphincter

The anal sphincter was seen to relax spontaneously, that is without association of a motor pattern in 24 instances observed across 10 of the 19 subjects. Hence 9 of the subjects had no independent anal sphincter relaxations. The anal sphincter was occasionally seen to relax rhythmically at 1 cpm as reported previously (13). The average percent anal sphincter relaxation during the independent relaxations (50.0%) was significantly lower compared with that of relaxations associated with motor patterns (*P* < 0.0001). Additionally, none of the independent relaxations reached 100%, while complete relaxation of the anal sphincter was observed in association with 12% of the HAPWs.

### Comparison Between HAPW Subgroups

#### Amplitude.

Transverse and descending HAPWs (*category 3*) had the highest average amplitude (109.3 mmHg), which was significantly higher than both *categories 1* (*P* < 0.0001) and *2* (*P* < 0.0001). *Categories 1* and *2* were also significantly different from one another (*P* = 0.0179), with proximal HAPWs having the lowest amplitude of all three.

#### Velocity.

The category with the highest amplitude HAPWs had the lowest mean velocity. *Category 3* was significantly lower than both *categories 1* (*P* = 0.0332) and *2* (*P* = 0.0076). *Categories 1* and *2* were not significantly different from each other.

#### HAPW Index.

Proximal continuing HAPWs (*category 2*) had the highest HAPW Index. It was significantly higher than both *categories 1* (*P* < 0.0001) and *3* (*P* = 0.0059). *Categories 1* and *3* were also significantly different from each other (*P* < 0.0001), with *category 1* having the lowest HAPW Index.

#### Site of origin and termination.

The majority of HAPWs terminated at the descending colon (66%), with another 6% terminating at the splenic flexure. Twenty-one percent of HAPWs propagated to the transverse colon, with an additional 2% terminating at the hepatic flexure. Five percent of the HAPWs entered the rectum, 1% of which were proximally originating, and the rest originated in the transverse or distal colon. There was no significant difference between the number of HAPWs, which originated in the proximal colon, compared with those which originated in the transverse/descending (51% and 49%, respectively). The proximally originating HAPWs did propagate a longer distance than the transverse-descending originating ones (27.0 cm and 23.8 cm respectively; *P* < 0.05). No significant difference in average anal sphincter relaxation was observed between any of the three categories.

### Response to Interventions

#### Baseline (90 min), N = 19 subjects.

Thirty-three HAPWs were observed during baseline, in eight individuals (Table 1) dominated by proximal a proximal HAPW followed by SPW (HAPW-SPWs) and transverse-descending HAPWs. The symbol maps show that isolated SPWs are the dominant motor pattern, as reported on previously (13, 15) (Fig. 4*A*). Only 16% of the subjects did not have any HAPW or SPW at baseline. The HAPW Index for baseline was 1,432 ± 215 mmHg·m·s, and its range was 175 to 4,549 mmHg·m·s (Fig. 6). The amplitude during baseline was 89.1 ± 4.9 mmHg, with a range of 79.1 to 99.1 mmHg. The velocity ranged between 0.61 cm/s and 0.80 cm/s, with an average of 0.71 ± 0.05 cm/s.

**Table 1.** HAPW categories and interventions

	Total HAPWs *N* = 19; *n* = 290	Proximal *Category 1*	Proximal Continuing *Category 2*	Transverse/Descending *Category 3*
*Baseline*				
Occurrence	*N* = 8; *n* = 33; *n*/*N* = 4.1; n/total *N* = 1.7	*N* = 2; *n* = 2	*N* = 5; *n* = 9	*N* = 6; *n* = 22
Amplitude, mmHg	89.1 ± 4.9 46.1–132.8	64.5 ± 15.4 49.1–79.9	68.9 ± 5.9 44.8–100.0	99.7 ± 5.7 47.9–136.4
Velocity, cm/s	0.71 ± 0.04† 0.32–1.1	0.64 ± 0.03 0.62–0.67	0.82 ± 0.10 0.34–1.2	0.66 ± 0.05 0.28–1.1
Index, mmHg·m·s	1,432 ± 215†† 175–4549	247 ± 83 164–330	1351 ± 430 286–4198	1573 ± 264 211–5063
*Proximal balloon distention (PBD)*				
Occurrence	*N* = 16; *n* = 45; *n*/*N* = 2.8; *n*/total *N* = 2.4	*N* = 0; *n* = 0	*N* = 4; *n* = 10	*N* = 16; *n* = 16
Amplitude, mmHg	104.0 ± 5.2* 41.4–166.8	N/A	123.9 ± 7.3 93.4–154.4	98.3 ± 6.0 38.0–174.8
Velocity, cm/s	0.49 ± 0.04†‡**## 0.32–1.1	N/A	0.38 ± 0.04 0.24–0.61	0.52 ± 0.05 0.15–1.2
Index, mmHg·m·s	2,973 ± 445††‡‡ 128–11,156	N/A	7,012 ± 988 3,984–12,670	1,819 ± 283 111–5,781
*Distal balloon distention (DBD)*				
Occurrence	*N* = 6; *n* = 12; *n*/*N* = 2; *n*/total *N* = 0.6	*N* = 1; *n* = 2	*N* = 5; *n* = 6	*N* = 2; *n* = 4
Amplitude, mmHg	90.9 ± 9.0 45.2–156.3	51.7 ± 6.3 45.4–58.0	86.4 ± 9.3 53.4–122.8	117.2 ± 13.5 05.7–156.3
Velocity, cm/s	1.4 ± 0.38 0.17–3.9	1.7 ± 0.52 1.2–2.2	1.4 ± 0.53 0.34–3.5	1.2 ± 0.92 0.17–3.9
Index, mmHg·m·s	1,667 ± 478 89–6269	93 ± 4 89–96	2,218 ± 839 875–6,269	1,626 ± 435 625–2,746
*Meal*				
Occurrence	*N* = 13; *n* = 48; *n*/*N* = 3.7; *n*/total *N* = 2.5	*N* = 3; *n* = 10	*N* = 6; *n* = 19	*N* = 8; *n* = 19
Amplitude, mmHg	77.2 ± 3.3*# 47.6–122.7	91.3 ± 8.7 51.2–125.7	65.3 ± 2.45 51.2–87.11	81.7 ± 5.5 43.2–125.9
Velocity, cm/s	0.77 ± 0.05‡ 0.44–1.6	0.87 ± 0.15 0.43–2.0	0.83 ± 0.09 0.41–1.7	0.65 ± 0.03 0.48–0.96
Index, mmHg·m·s	950 ± 130‡‡***### 91–2,768	329 ± 74 61–708	1375 ± 232 253–3,761	851 ± 183 71–2,660
*Oral prucalopride*				
Occurrence	*N* = 11; *n* = 53; *n*/*N* = 4.8; *n*/total *N* = 2.8	*N* = 8; *n* = 21	*N* = 4; *n* = 15	*N* = 4; *n* = 17
Amplitude, mmHg	94.7 ± 6.5 49.5–195.1	66.1 ± 5.5 40.9–155.8	77.8 ± 4.5 55.6–107.9	144.9 ± 11.4 61.3–200.7
Velocity, cm/s	0.69 ± 0.04** 0.21–1.3	0.67 ± 0.05 0.30–1.3	0.77 ± 0.09 0.29–1.5	0.63 ± 0.06 0.29–1.2
Index, mmHg·m·s	2,624 ± 460*** 130–11,969	479 ± 122 125–2,295	3,502 ± 831 480–11,952	4,500 ± 985 135–13,044
*Rectal bisacodyl*				
Occurrence	*N* = 12; *n* = 59; *n*/*N* = 4.9; *n*/total *N* = 3.1	*N* = 5; *n* = 16	*N* = 5; *n* = 16	*N* = 8; *n* = 27
Amplitude, mmHg	103.2 ± 5.2# 50.9–189.7	80.5 ± 6.0 50.7–148.7	97.6 ± 7.8 71.0–200.7	120.0 ± 8.5 49.3–196.2
Velocity, cm/s	0.78 ± 0.09## 0.29–1.7	1.2 ± 0.28 0.23–5.0	0.73 ± 0.06 0.29–1.2	0.63 ± 0.05 0.27–1.4
Index, mmHg·m·s	2,337 ± 436### 86–10,470	325 ± 74 58–1,300	5,232 ± 1,101 1,210–16,416	1,813 ± 467 97–9,282

Values are expressed as averages ± SE values and 95th percentile normal ranges. *N* refers to number of subjects, while *n* refers to the number of HAPWs.

* *P* = 0.0005;

# *P* = 0.0007;

† *P* = 0.0069;

‡ *P* = 0.0005;

** *P* = 0.0036;

## *P* = 0.0304;

†† *P* = 0.0317;

‡‡ *P* = 0.0008;

*** *P* = 0.0108;

### *P* = 0.0371.

**Fig. 4.**
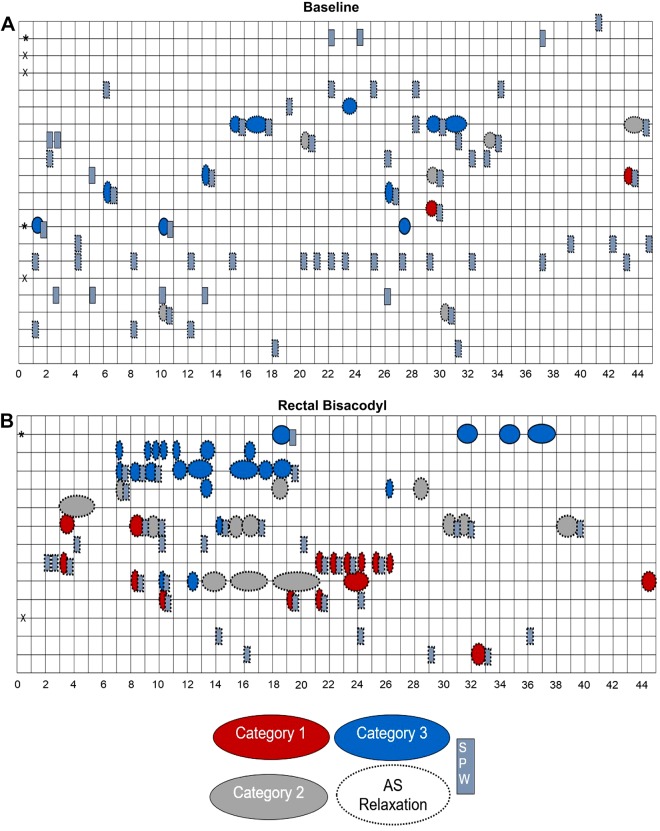
Symbol maps of baseline (*A*) and rectal bisacodyl injection (*B*). Symbol map of baseline includes the second half of the 90-min baseline recording, 45 min before the subsequent intervention. Each row represents a single volunteer. X represents a lack of response; * represents no visualization of the anal sphincter as a consequence of all sensors being oral to the sphincter. A dotted outline around a symbol indicates that the motor pattern is associated with anal sphincter relaxation. There was a relatively uniform response across the subjects, dominated by simultaneous pressure waves (SPWs) during baseline. Rectal bisacodyl induced an overall strong response dominated by high-amplitude propagating pressure waves (HAPWs), with great variability in the type of HAPWs that were observed.

#### Proximal balloon distention (20 min), N = 19 subjects.

**Fig. 5.**
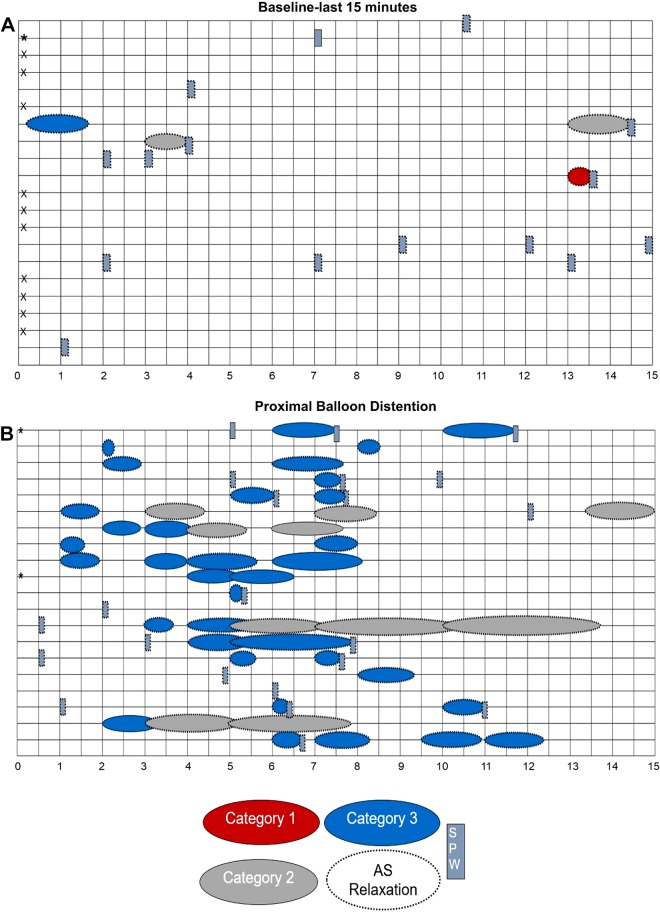
Symbol maps of last 15 min of baseline (*A*) and proximal balloon distention (*B*). Each row represents a single volunteer. X indicates a lack of response; *represents no visualization of the anal sphincter during high-resolution colonic manometry (HRCM). A dotted outline around a symbol indicates association of a motor pattern with anal sphincter relaxation. During proximal balloon distention, all subjects showed a response, and it was dominated by high-amplitude propagating pressure waves (HAPWs), most of which originated in the transverse/descending colon.

**Fig. 6.**
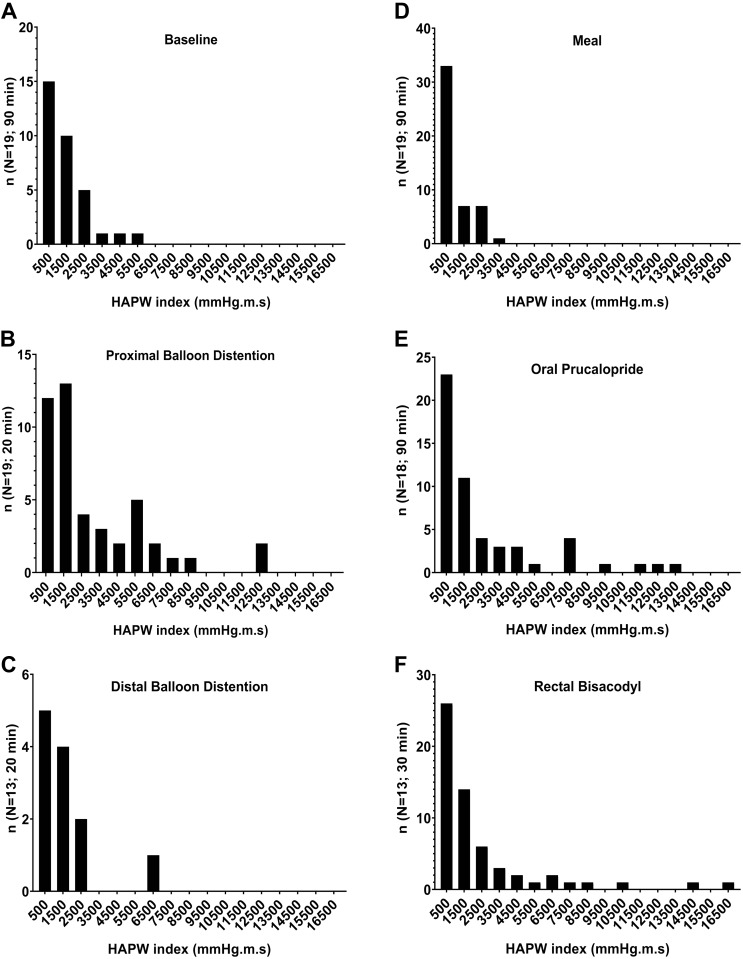
High-amplitude propagating pressure waves (HAPW) Indexes separated based on interventions. Shown are baseline (*A*), proximal balloon distention (*B*), distal balloon distention (*C*), meal (*D*), oral prucalopride (*E*), and rectal bisacodyl (*F*; *N* = 19 subjects, *n* = 290 HAPWs). Center of bin is shown on *x*-axis.

Forty-four HAPWs were observed during proximal balloon distention, in all individuals (Table 1) (Fig. 5), dominated by HAPWs from *categories 2* and *3*. The average HAPW Index from proximal balloon distention was 2,973 ± 445 mmHg·m·s, and its range was 128 to 11,156 mmHg·m·s (Fig. 6). The mean HAPW amplitude during the intervention was 105.2 ± 5.1 mmHg with a range of 94.8 to 115.5 mmHg. The velocity ranged between 0.40 and 0.55 cm/s with a mean of 0.47 ± 0.04 cm/s.

#### Meal response (90 min), N = 19 subjects.

Forty-nine HAPWs were observed after meal intake (Table 1) in 13 subjects, showing a large intersubject variability in the generation of the HAPWs and also in the time they appeared after intake of the meal (Supplemental Fig. S1, www.doi.org/10.6084/m9.figshare.11831346.v1; all supplemental material may be found at this site.). Although the exact timing of the gastrocolonic reflex is difficult to determine because it cannot be excluded that some HAPWs would have appeared even without the meal, the start of the gastrocolonic reflex took an average of 24.1 ± 4.6 min, with a range of 7 to 62 min. Hence, to ensure the reflex has materialized, an observation time of at least 60 min from beginning of meal intake is essential. Thirty-two percent of healthy subjects did not generate HAPWs but did respond to the meal with SPWs (13, 15) (Supplemental Fig. S1). The mean amplitude of HAPWs during this intervention was 77.2 ± 3.3 mmHg, and the mean velocity was 0.77 ± 0.05 cm/s. The average HAPW Index was 950 ± 130 mmHg·m·s, and the normal range was 91 to 2,768 mmHg·m·s (Fig. 6).

#### Oral prucalopride (90 min), N = 18 subjects.

Fifty-two HAPWs were observed in 10 individuals after oral prucalopride intake (Table 1) (Supplemental Fig. S2). The response to prucalopride (4 mg) was variable both in onset time and type of response. The average HAPW Index for this intervention was 2,624 ± 460 mmHg·m·s, and the normal range was 130 to 11,969 mmHg·m·s (Fig. 6). The amplitude of HAPWs during this intervention was 94.7 ± 6.5 mmHg, ranging between 81.7 and 107.7 mmHg. The average propagating velocity was 0.69 ± 0.04 cm/s, and it ranged between 0.61 to 0.76 cm/s.

#### Rectal bisacodyl (20 min), N = 13 subjects.

**Fig. 7.**
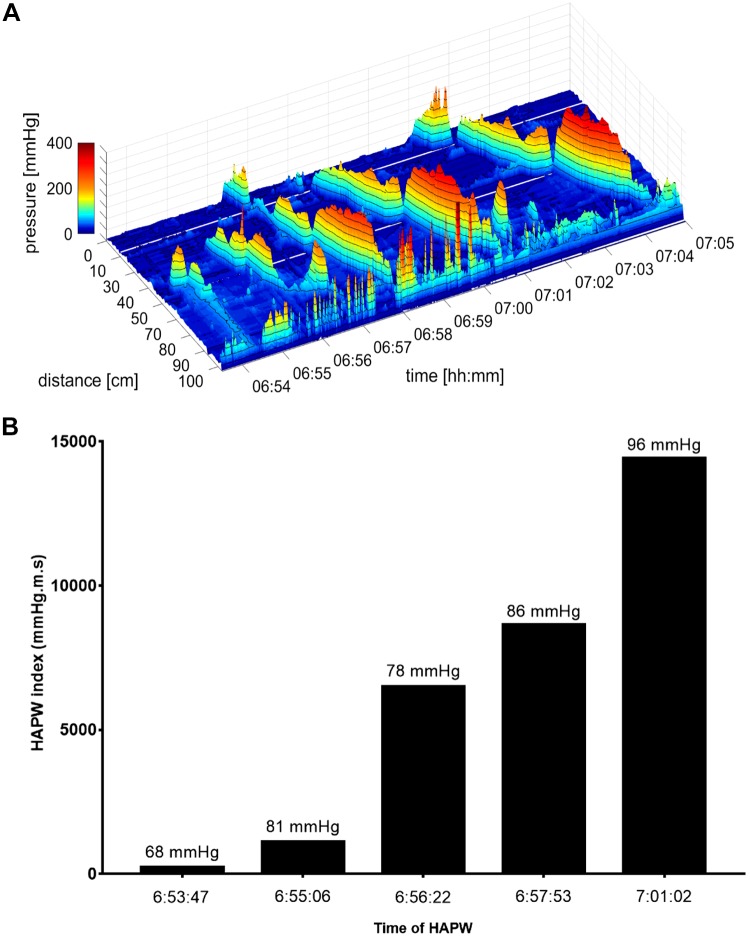
HAPW Indexes of individual HAPWs in response to rectal bisacodyl injection. A gradual increase in the HAPW Index can be observed that corresponds with the increase in excitation caused by the intervention, and the size and propagation length of the HAPWs.

Fifty-eight HAPWs were observed in 12 individuals in response to rectal bisacodyl (Table 1). Rectally administered bisacodyl induced an early response and the greatest number of HAPWs, belonging to each of the three categories (Table 1). This was the only intervention in which pancolonic HAPWs were observed, entering the rectum. The symbol map for this intervention shows a large variability with regard to the type of HAPWs that can be observed (Fig. 4). Only a single subject had no response to this intervention, and only two responded with SPWs alone. Most subjects responded within the first 10 min of administration (Fig. 4); only two subjects exceeded that time by a few minutes. HAPWs during this intervention had an amplitude of 104.1 ± 5.2 mmHg, ranging between 93.8 and 114.5 mmHg. The propagating velocity ranged between 0.64 and 0.82 cm/s, with an average of 0.73 ± 0.05 cm/s. Rectal bisacodyl HAPWs had an HAPW Index of 2,337 ± 436 mmHg·m·s, with a range of 86 to 10,470 mmHg·m·s (Fig. 6). Figure 7 shows a response to bisacodyl (10 mg), illustrating the gradual increase in excitation of the musculature represented by a gradual increase in the HAPW Index.

### Comparison Between Interventions

Proximal balloon distention was the intervention during which the highest average HAPW amplitude was observed. The second-highest amplitude was observed in response to rectal bisacodyl (Table 1). The meal was the intervention that induced the lowest mean amplitude HAPWs. The opposite was true with regard to velocity, where the meal was observed to have induced HAPWs at the highest velocity on average, and proximal balloon distention induced the lowest velocity HAPWs. In addition to amplitude, proximal balloon distention induced HAPWs with the highest HAPW Index, and similarly, the meal showed the lowest HAPW Index (Table 1).

### Assessing Normal HAPW Activity

Protocols with only baseline and a meal, or baseline and rectal bisacodyl injection have a high probability (6 of 19) of exhibiting nonresponding subjects. A meal, baseline, and rectal bisacodyl injection revealed 5 of 19 nonresponders (Table 2). Proximal balloon distention was observed to be a superior intervention with a low probability of nonresponders, even when used alone. When proximal balloon distention was used with meal or rectal bisacodyl, each of these combinations of interventions only had 1 volunteer who did not respond with HAPWs, although the occurrence of HAPWs oral to the most proximal sensor cannot be excluded. A combination of baseline, proximal balloon distention, and meal was able to induce HAPWs with the highest amplitude and HAPW Index. Combining baseline, proximal balloon distention, meal, and rectal bisacodyl injection gives a high likelihood of observing HAPWs, with only 1 nonresponder (Table 2).

**Table 2.** Response to combination of interventions

Intervention	Baseline + PBD	Baseline + Meal	Baseline + RB	Baseline + PBD+ Meal	Baseline + Meal + RB	Baseline + PBD + RB	Baseline + PBD + Meal + RB
HAPWs/ subject	4.2 ± 0.73*	4.5 ± 1.1	5.1 ± 1.2	6.9 ± 1.1	7.7 ± 1.7	7.4 ± 1.1	9.6 ± 1.3*
Average amplitude, mmHg	97.7 ± 3.7† 45.3–151.3	82.1 ± 2.8†#‡** 46.8–125.9	98.2 ± 3.8# 49.0–181.0	89.9 ± 2.8 45.8–147.8	91.0 ± 2.9 49.2–175.8	100.1 ± 3.1‡ 48.6–180.2	94.2 ± 2.5** 48.9–171.8
Average Index, mmHg·m·s	2,321 ± 285†† 153–7,328	1,146 ± 119††‡‡*** 152–3,289	2,012 ± 293 153–7,894	1,799 ± 192 151–5,855	1,648 ± 202 132–6,315	2,328 ± 247‡‡ 149–8,521	1,970 ± 191*** 137–7,029
Number of HAPW nonresponders	2	6	6	1	5	1	1

Average amplitude and index are presented as means ± SE. Symbols are noted at the two values that are compared:

* *P* = 0.0185;

† *P* = 0.0174;

# *P* = 0.0146;

‡ *P* = 0.0005;

** *P* = 0.0280;

†† *P* = 0.0043;

‡‡ *P* = 0.0005;

*** *P* = 0.0055.

### Symptoms and Other Events

**Fig. 8.**
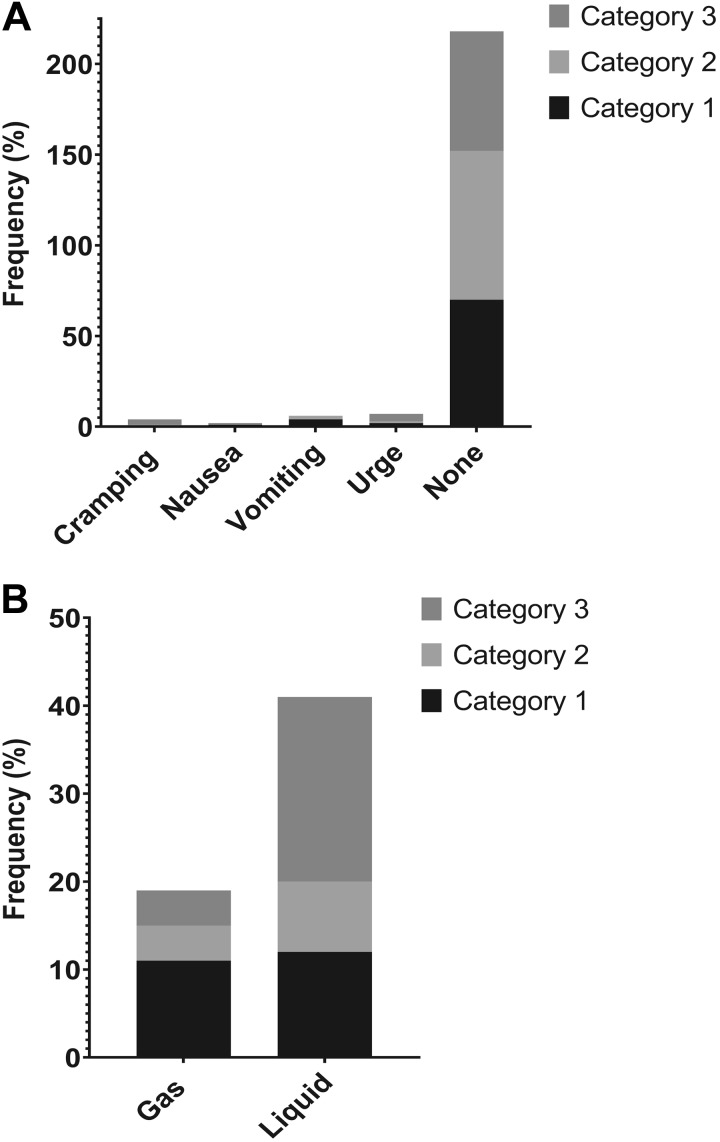
Symptoms and transit events associated with high-amplitude propagating pressure waves (HAPWs). *A*: most HAPWs were not associated with any reported symptoms. *B*: frequency of HAPW-associated liquid expulsion was similar across all 4 categories of HAPWs. Gas expulsion was most commonly reported with HAPWs starting in the proximal and terminating in the descending colon. (*N* = 19 subjects, *n* = 290 HAPWs).

HAPWs in all three categories were most commonly not associated with symptoms or gas or liquid expulsion (Fig. 8*A*). HAPW-associated liquid expulsion was seen most commonly in transverse/descending HAPWs. Gas expulsion was most commonly reported with HAPWs from *category 1* (Fig. 8*B*). Urge to defecate was the most common symptom, it was most often reported with transverse/distal HAPWs. Nausea was only reported with 2% of HAPWs and was never associated with those from *category 2*. In 3 of the 19 subjects, vomiting occurred with *category 1* and *category 2* HAPWs, but not with transverse/descending HAPWs; 56% of vomiting episodes occurred in the 90-min period after oral prucalopride.

## DISCUSSION

### Overall Features of HAPWs

Here, we present a comprehensive assessment of the HAPW using 84 sensors, 1 cm apart, throughout the colon. We show that HAPWs in the healthy adult (18 yr old and older) can be restricted to the proximal colon, can start and terminate in the transverse and descending colon, and are rarely pan-colonic. Previous manometric data in adults already indicated that HAPWs do not necessarily progress as HAPWs toward the rectum (6), but this contrasts with studies in the pediatric population, where only pancolonic HAPWs are considered normal, with the understanding that no data are available for healthy children (47). In the United States, pediatric patients include ages 18–20 yr. In the present study, three subjects were 20 yr of age, and they showed all categories of HAPWs. Here, we show that 52% of HAPWs transform into a simultaneous pressure wave, and in this way, they reach the rectum. Therefore, we recommend that the assessment of HAPWs should include the recognition of HAPW-SPWs.

### Colo-Anal Reflex

HAPWs are associated with anal sphincter relaxation, defined as the colo-anal reflex (19, 34, 48, 49, 52). Here, we show that this relaxation occurred with all categories of HAPW and was, on average, 66% of its baseline anal pressure. We also show that the relaxation often amounts to 100%, indicating that the relaxation involves the external anal sphincter. Hence, the relaxation of the external anal sphincter involves spinal autonomic nerves likely acting on the efferent nerves in Onuf’s nucleus (8, 10). The colo-anal reflex is probably an essential component of defecation, involving autonomic sacral neural pathways (30). It is not assessed by ano-rectal manometry, which tests the recto-anal inhibitory reflex (RAIR) in response to rectal balloon distention. The RAIR only involves enteric nitrergic relaxation of the internal anal sphincter (42, 48), and usually 24 mmHg of resting pressure remains during balloon distention (47). Consistently, in children with disrupted continuity of the colon, which abolishes RAIR, the colo‐anal reflex was preserved, indicating that it is mediated by a different pathway from the RAIR, likely an extrinsic neural pathway (49). In dogs, anal relaxation upon proximal colon distention was mediated by sympathetic nerves (16). Anal sphincter relaxation also occurs in response to the simultaneous pressure wave (13, 15, 20) (see the symbol maps in Figs. 4 and 5, Supplemental Figs. S1 and S2); hence, the term colo-anal reflex should be defined as the autonomous relaxation of the anal sphincters in response to propulsive colonic motor patterns.

### When Should HAPWs Be Described as Low-Amplitude Propagating Pressure Waves

Here, we show that the amplitude of HAPWs in healthy subjects had an individual average value >50 mmHg, and the lowest maximal pressure was 88 mmHg; hence, we suggest that in patients with HAPWs <50 mmHg, the HAPW may be of insufficient force, and the motor pattern should be referred to as low-amplitude propagating pressure wave (LAPW). This was also proposed by Bassotti et al. (2, 3), although a consensus report suggested a cut-off of 75 mmHg based on low-resolution manometry (11) and based on studies that used only the maximum amplitude of a single measuring point of the HAPWs. We agree with Bampton et al. (1) and Bassotti et al. (4) that the activities are on a continuum and that HAPWs and LAPWs should not really be seen as different motor patterns. LAPWs should be seen as possibly inefficient HAPWs. With regard to velocity of the HAPWs, we observed them to range between 0.2 cm/s and 2.2 cm/s, consistent with values obtained from previous studies (18, 23, 34). Motor patterns of low amplitude have been associated with myogenic dysfunction (33), but it is also possible that insufficient neural excitation is the underlying dysfunction.

### The Creation of Symbol Maps

When assessing normal occurrence of HAPWs under baseline conditions or in response to a meal, it is customary to present the average value of the number of HAPWs, as well as a normal range. However, decisions about abnormality should not be made solely by comparing features of a patient’s HAPWs with average values from healthy persons. Here, we introduce the symbol map to give an overview of baseline activity and responses to interventions of all subjects, with details about the HAPW category, their length of propagation, their association with SPWs and anal sphincter relaxation, and the time they occur relative to the start of the intervention. The dramatic development of HAPWs in response to balloon distention (Fig. 5*B*) and bisacodyl injection becomes immediately obvious (Fig. 4*B*), but the variability in response to stimuli in healthy subjects is also clear. This large variability is what makes diagnosing colon dysmotility more difficult compared with esophageal dysmotility.

### A Comprehensive Quantitative Assessment, the HAPW Index

The present study introduces the HAPW Index as a quantitative measure of the strength of the HAPW. In clinical assessments of colonic motility thus far, only the HAPW amplitude and velocity are quantified. Figure 7 shows clearly that the HAPW Index better represents the strength of the HAPW compared with the amplitude alone. We show that the average HAPW Index centers around 1,400 mmHg·m·s at baseline, ~950 mmHg·m·s after a meal, ~2,600 mmHg·m·s after oral prucalopride, ~3,000 mmHg·m·s during proximal balloon distention, and ~2,300 mmHg·m·s in response to rectal bisacodyl. It is clear from these data that a single index value does not appear to be useful; the index should be linked to baseline or a specific intervention.

### Development of Optimal Stimulus Parameters

We show here that rectal bisacodyl and proximal balloon distention are stimuli that have a high chance of evoking all types of HAPWs. These stimuli are rarely performed but are highly effective. Rectal bisacodyl evoked HAPWs that started in the proximal colon after ~10 min. Hence, bisacodyl will activate extrinsic sensory nerves that communicate with the spinal cord neurons, which ultimately evoke vagal responses to initiate proximal HAPWs (39, 54). A positive response to rectal bisacodyl confirms intactness of critical neural reflexes. When a HAPW develops, it also shows normal colonic musculature and enteric neural circuits. In patients who do not have spontaneous bowel movements, rectal bisacodyl may evoke HAPWs; hence, although bisacodyl activates physiological reflexes, it is a powerful pharmacological substance that does not necessarily mimic a physiological rectal stimulation; nevertheless, a positive response shows that the spinal and vagal innervation, as well as the communication between autonomic nerves and the colon are present and intact (12). Rectal stimulation will become more important in the future since solid-state catheters and fiber-optic catheters do not have the ability to deliver a stimulus to the proximal colon, a stimulus that was routinely given using water-perfused catheters. Although both proximal and rectal bisacodyl can induce HAPWs that start in the proximal colon, a different mechanism of action may underlie it, as the proximal and distal colon is predominantly innervated by the vagus and sacral nerves, respectively (21) (13, 39, 54). Although proximal bisacodyl can evoke HAPWs, rectal bisacodyl may be more relevant for the testing of the rectal reflex to initiate HAPWs. Our data are consistent with early studies from Preston and Lennard-Jones (40), who looked at bisacodyl instilled within the recto-sigmoid area, and found that in healthy controls, there was a marked increase in anally progressing propagating waves. We show here that proximal balloon distention is a very good stimulus to evaluate if the colon is capable of generating propulsive motor patterns. The stimulus evoked all three categories of strong HAPWs that were of a high amplitude, but a slower velocity compared with baseline (Table 1), which, on the basis of other studies, are likely able to propel content (18). There were only two subjects who did not respond to proximal balloon distention with HAPWs, but they did respond with SPWs (Table 2, Fig. 5*B*). Proximal balloon distention activates sensory receptors in the proximal colon that can initiate motor patterns starting proximal to the stimulus, likely mediated by both extrinsic vagal pathways, as well as the enteric nervous system (56). It likely imitates food entering the colon, and in our study, it evoked mostly HAPWs with origin in the transverse or descending colon. Similarly, Kamm et al. (29) observed HAPWs in response to proximal balloon distention that expelled isotope, although these HAPWs entered the rectum, which never happened in our studies.

It may be useful to test the response to a meal, although it has a lower chance of evoking HAPWs compared with the above-mentioned stimuli. A colonic response to a meal signifies the gastro-colonic reflex, a vagally mediated “awakening” of the colon (1, 46, 51). The present study shows that a meal can evoke HAPWs, which have amplitudes on the lower end of the spectrum, but with a higher velocity. It is also evident that the response is highly variable with many healthy subjects showing no or a late response to the intervention and others showing an increase in SPWs but no HAPWs, as shown in the symbol maps. We defined the presence of the gastrocolonic reflex as an increase in propulsive motor patterns compared with baseline following a meal (50). Considering our observation that healthy subjects may not exhibit a response to meal, in patients, a positive response, whether it is HAPWs or SPWs, suggests intactness of vagal innervation. However, no response, by itself, does not necessarily identify pathophysiology.

On the basis of a previous study (31), we hypothesized that oral prucalopride, once entering the stomach, would activate the numerous 5-HT_4_ receptors on the luminal surface of epithelial cells (55), releasing 5HT from enterochromaffin cells to activate vagal sensory nerves that might lead to a gastro-colonic reflex. In the present study, 5 of 17 subjects who took oral prucalopride showed a HAPW response within 15 min, possibly due to this gastro-colonic reflex. Since the response was not consistently observed, the clinical value of giving prucalopride as a diagnostic tool during HRCM is questionable. HAPWs generated after prucalopride intake did show a significantly higher amplitude compared with the meal response; in addition, they were most commonly associated with vomiting compared with other interventions. This may be related to prucalopride’s stimulating effects of 5-HT_4_ vagal afferents, which send signals to stimulate the vomiting center in the brain (53).

On the basis of our experience, an optimal protocol to assess colon function, including the gastrocolonic reflex is baseline period, a meal, proximal balloon distention, and rectal bisacodyl injection. If the only objective is to observe HAPWs and there are time constraints, then proximal balloon distention and/or rectal bisacodyl injection may be sufficient.

## GRANTS

J. D. Huizinga received a Canadian Foundation for Innovation John Evans Leadership grant for the equipment used in this study. Operating funds were obtained from the Hamilton Academic Health Sciences Organization to E. Ratcliffe and from the Canadian Institutes of Health Research (152942) to J. D. Huizinga The Farncombe Family Digestive Health Research Institute provided partial salary support for J. -H. Chen and S. P. Parsons.

## DISCLOSURES

No conflicts of interest, financial or otherwise, are declared by the authors.

## AUTHOR CONTRIBUTIONS

J.H.C. and J.D.H. conceived and designed research; N.M., J.D.H., and J.H.C. performed experiments; N.M., S.P.P., J.D.H., and J.H.C. analyzed data; N.M., S.P.P., J.D.H., and J.C. interpreted results of experiments; N.M., J.D.H., and J.C. prepared figures; N.M., S.P.P., J.D.H., and J.H.C. drafted manuscript; N.M., S.P.P., E.R., J.D.H., and J.H.C. edited and revised manuscript; N.M., S.P.P., E.R., J.D.H., and J.H.C. approved final version of manuscript.

## References

[1] Bampton PA, Dinning PG, Kennedy ML, Lubowski DZ, deCarle D, Cook IJ Spatial and temporal organization of pressure patterns throughout the unprepared colon during spontaneous defecation. Am J Gastroenterol 95: 1027–1035, 2000. doi:10.1111/j.1572-0241.2000.01839.x. 10763955

[2] Bassotti G, Chistolini F, Marinozzi G, Morelli A Abnormal colonic propagated activity in patients with slow transit constipation and constipation-predominant irritable bowel syndrome. Digestion 68: 178–183, 2003. doi:10.1159/000075554. 14671425

[3] Bassotti G, de Roberto G, Castellani D, Sediari L, Morelli A Normal aspects of colorectal motility and abnormalities in slow transit constipation. World J Gastroenterol 11: 2691–2696, 2005. doi:10.3748/wjg.v11.i18.2691. 15884105 PMC4305899

[4] Bassotti G, Iantorno G, Fiorella S, Bustos-Fernandez L, Bilder CR Colonic motility in man: features in normal subjects and in patients with chronic idiopathic constipation. Am J Gastroenterol 94: 1760–1770, 1999. doi:10.1111/j.1572-0241.1999.01203.x. 10406232

[5] Bharucha AE, Brookes SJH. Neurophysiologic mechanisms of human large intestinal motility. In: Physiology of the Gastrointestinal Tract. New York: Elsevier, 2018, 517–564.

[6] Bharucha AE High amplitude propagated contractions. Neurogastroenterol Motil 24: 977–982, 2012. doi:10.1111/nmo.12019. 23057554 PMC3471560

[7] Bharucha AE, Anderson B, Bouchoucha M More movement with evaluating colonic transit in humans. Neurogastroenterol Motil 31: e13541, 2019. doi:10.1111/nmo.13541. 30681255 PMC6362846

[8] Broens PM, Penninckx FM, Ochoa JB Fecal continence revisited: the anal external sphincter continence reflex. Dis Colon Rectum 56: 1273–1281, 2013. doi:10.1097/DCR.0b013e3182a42d16. 24105003

[9] Brookes SJ, Dinning PG, Gladman MA Neuroanatomy and physiology of colorectal function and defaecation: from basic science to human clinical studies. Neurogastroenterol Motil 21, Suppl 2: 9–19, 2009. doi:10.1111/j.1365-2982.2009.01400.x. 19824934

[10] Callaghan B, Furness JB, Pustovit RV Neural pathways for colorectal control, relevance to spinal cord injury and treatment: a narrative review. Spinal Cord 56: 199–205, 2018. doi:10.1038/s41393-017-0026-2. 29142293

[11] Camilleri M, Bharucha AE, di Lorenzo C, Hasler WL, Prather CM, Rao SS, Wald A American Neurogastroenterology and Motility Society consensus statement on intraluminal measurement of gastrointestinal and colonic motility in clinical practice. Neurogastroenterol Motil 20: 1269–1282, 2008. doi:10.1111/j.1365-2982.2008.01230.x. 19019032

[12] Chen J-H, Huizinga JD High-pressure tactic: colonic manometry in chronic constipation. Dig Dis Sci 63: 2820–2822, 2018. doi:10.1007/s10620-018-5160-y. 29881907

[13] Chen J-H, Parsons SP, Shokrollahi M, Wan A, Vincent AD, Yuan Y, Pervez M, Chen WL, Xue M, Zhang KK, Eshtiaghi A, Armstrong D, Bercik P, Moayyedi P, Greenwald E, Ratcliffe EM, Huizinga JD Characterization of simultaneous pressure waves as biomarkers for colonic motility assessed by high-resolution colonic manometry. Front Physiol 9: 1248, 2018. doi:10.3389/fphys.2018.01248. 30294277 PMC6159752

[14] Chen J-H, Pervez M, Hanman AA, Shokrollahi M, Parsons S, Ratcliffe E, Huizinga JD Different types of simultaneous pressure waves in the human colon: biomarkers for gas transit and colon function assessment. Neurogastroenterol Motil 30-S1, Aug. 6, 2018. doi:10.1111/nmo.13422.

[15] Chen J-H, Yu Y, Yang Z, Yu W-Z, Chen WL, Yu H, Kim MJ, Huang M, Tan S, Luo H, Chen J, Chen JD, Huizinga JD Intraluminal pressure patterns in the human colon assessed by high-resolution manometry. Sci Rep 7: 41436, 2017. doi:10.1038/srep41436. 28216670 PMC5316981

[16] Chen J-H, Sallam HS, Lin L, Chen JD Colorectal and rectocolonic reflexes in canines: involvement of tone, compliance, and anal sphincter relaxation. Am J Physiol Regul Integr Comp Physiol 299: R953–R959, 2010. doi:10.1152/ajpregu.00439.2009. 20554930 PMC2944431

[17] Conklin JL Evaluation of esophageal motor function with high-resolution manometry. J Neurogastroenterol Motil 19: 281–294, 2013. doi:10.5056/jnm.2013.19.3.281. 23875094 PMC3714405

[18] Cook IJ, Furukawa Y, Panagopoulos V, Collins PJ, Dent J Relationships between spatial patterns of colonic pressure and individual movements of content. Am J Physiol Gastrointest Liver Physiol 278: G329–G341, 2000. doi:10.1152/ajpgi.2000.278.2.G329. 10666058

[19] Corsetti M, Costa M, Bassotti G, Bharucha AE, Borelli O, Dinning P, Di Lorenzo C, Huizinga JD, Jimenez M, Rao SS, Spiller R, Spencer N, Lentle R, Pannemans J, Thys A, Benninga M, Tack J First translational consensus on terminology and definitions of colonic motility in animals and humans studied by manometric and other techniques. Nat Rev Gastroenterol Hepatol 16: 559–579, 2019. doi:10.1038/s41575-019-0167-1. 31296967 PMC7136172

[20] Corsetti M, Pagliaro G, Demedts I, Deloose E, Gevers A, Scheerens C, Rommel N, Tack J Pan-colonic pressurizations associated with relaxation of the anal sphincter in health and disease: a new colonic motor pattern identified using high-resolution manometry. Am J Gastroenterol 112: 479–489, 2017. doi:10.1038/ajg.2016.341. 27596695

[21] Dapoigny M, Cowles VE, Zhu YR, Condon RE Vagal influence on colonic motor activity in conscious nonhuman primates. Am J Physiol Gastrointest Liver Physiol 262: G231–G236, 1992. doi:10.1152/ajpgi.1992.262.2.G231. 1539658

[22] De Schryver AM, Samsom M, Smout AI Effects of a meal and bisacodyl on colonic motility in healthy volunteers and patients with slow-transit constipation. Dig Dis Sci 48: 1206–1212, 2003. doi:10.1023/A:1024178303076. 12870774

[23] Dinning PG, Bampton PA, Andre J, Kennedy ML, Lubowski DZ, King DW, Cook IJ Abnormal predefecatory colonic motor patterns define constipation in obstructed defecation. Gastroenterology 127: 49–56, 2004. doi:10.1053/j.gastro.2004.03.066. 15236171

[24] Dinning PG, Wiklendt L, Maslen L, Gibbins I, Patton V, Arkwright JW, Lubowski DZ, O’Grady G, Bampton PA, Brookes SJ, Costa M Quantification of in vivo colonic motor patterns in healthy humans before and after a meal revealed by high-resolution fiber-optic manometry. Neurogastroenterol Motil 26: 1443–1457, 2014. doi:10.1111/nmo.12408. 25131177 PMC4438670

[25] Grider JR, Kuemmerle JF, Jin JG 5-HT released by mucosal stimuli initiates peristalsis by activating 5-HT4/5-HT1p receptors on sensory CGRP neurons. Am J Physiol Gastrointest Liver Physiol 270: G778–G782, 1996. doi:10.1152/ajpgi.1996.270.5.G778. 8967488

[26] Herregods TV, Roman S, Kahrilas PJ, Smout AJ, Bredenoord AJ Normative values in esophageal high-resolution manometry. Neurogastroenterol Motil 27: 175–187, 2015. doi:10.1111/nmo.12500. 25545201

[27] Kahrilas PJ Esophageal motor disorders in terms of high-resolution esophageal pressure topography: what has changed? Am J Gastroenterol 105: 981–987, 2010. doi:10.1038/ajg.2010.43. 20179690 PMC2888528

[28] Kahrilas PJ, Bredenoord AJ, Fox M, Gyawali CP, Roman S, Smout AJ, Pandolfino JE, International; International High Resolution Manometry Working Group The Chicago Classification of Esophageal Motility Disorders, v3.0. Neurogastroenterol Motil 27: 160–174, 2015. doi:10.1111/nmo.12477. 25469569 PMC4308501

[29] Kamm MA, van der Sijp JR, Lennard-Jones JE Observations on the characteristics of stimulated defaecation in severe idiopathic constipation. Int J Colorectal Dis 7: 197–201, 1992. doi:10.1007/BF00341220. 1293240

[30] Knowles CH, Scott SM, Lunniss PJ Slow transit constipation: a disorder of pelvic autonomic nerves? Dig Dis Sci 46: 389–401, 2001. doi:10.1023/A:1005665218647. 11281190

[31] Li H, Yu Y, Yang Z, Yu W, Zhao L, Tan S, Luo H, Chen J-H, Huizinga JD High-resolution manometry changes diagnosis and treatment of patient with chronic constipation. Neurogastroenterol Motil 26, Suppl 1: 55–56, 2014.

[32] Lin Z, Roman S, Pandolfino JE, Kahrilas PJ Automated calculation of the distal contractile integral in esophageal pressure topography with a region-growing algorithm. Neurogastroenterol Motil 24: e4–e10, 2012. doi:10.1111/j.1365-2982.2011.01795.x. 21951921 PMC3608518

[33] Malagelada C, Karunaratne TB, Accarino A, Cogliandro RF, Landolfi S, Gori A, Boschetti E, Malagelada JR, Stanghellini V, Azpiroz F, De Giorgio R Comparison between small bowel manometric patterns and full-thickness biopsy histopathology in severe intestinal dysmotility. Neurogastroenterol Motil 30: e13219, 2018. doi:10.1111/nmo.13219. 28941004

[34] Malcolm A, Camilleri M Coloanal motor coordination in association with high-amplitude colonic contractions after pharmacological stimulation. Am J Gastroenterol 95: 715–719, 2000. doi:10.1111/j.1572-0241.2000.01840.x. 10710063

[35] Narducci F, Bassotti G, Gaburri M, Morelli A Twenty four hour manometric recording of colonic motor activity in healthy man. Gut 28: 17–25, 1987. doi:10.1136/gut.28.1.17. 3817580 PMC1432711

[36] Pandolfino JE, Ghosh SK, Rice J, Clarke JO, Kwiatek MA, Kahrilas PJ Classifying esophageal motility by pressure topography characteristics: a study of 400 patients and 75 controls. Am J Gastroenterol 103: 27–37, 2008. doi:10.1111/j.1572-0241.2007.01532.x. 17900331

[37] Pandolfino JE, Kahrilas PJ AGA technical review on the clinical use of esophageal manometry. Gastroenterology 128: 209–224, 2005. doi:10.1053/j.gastro.2004.11.008. 15633138

[38] Pervez M, Ratcliffe E, Parsons SP, Chen J-H, Huizinga JD The cyclic motor patterns in the human colon. Neurogastroenterol Motil 00: e13807, 2020. doi:10.1111/nmo.13807. 32124528

[39] Powley TL, Hudson CN, McAdams JL, Baronowsky EA, Phillips RJ Vagal intramuscular arrays: the specialized mechanoreceptor arbors that innervate the smooth muscle layers of the stomach examined in the rat. J Comp Neurol 524: 713–737, 2016. doi:10.1002/cne.23892. 26355387 PMC4784687

[40] Preston DM, Lennard-Jones JE Pelvic motility and response to intraluminal bisacodyl in slow-transit constipation. Dig Dis Sci 30: 289–294, 1985. doi:10.1007/BF01403835. 3979235

[41] Proano M, Camilleri M, Phillips SF, Brown ML, Thomforde GM Transit of solids through the human colon: regional quantification in the unprepared bowel. Am J Physiol Gastrointest Liver Physiol 258: G856–G862, 1990. doi:10.1152/ajpgi.1990.258.6.G856. 2360632

[42] Rao SS, Benninga MA, Bharucha AE, Chiarioni G, Di Lorenzo C, Whitehead WE ANMS-ESNM position paper and consensus guidelines on biofeedback therapy for anorectal disorders. Neurogastroenterol Motil 27: 594–609, 2015. doi:10.1111/nmo.12520. 25828100 PMC4409469

[43] Rao SS, Camilleri M, Hasler WL, Maurer AH, Parkman HP, Saad R, Scott MS, Simren M, Soffer E, Szarka L Evaluation of gastrointestinal transit in clinical practice: position paper of the American and European Neurogastroenterology and Motility Societies. Neurogastroenterol Motil 23: 8–23, 2011. doi:10.1111/j.1365-2982.2010.01612.x. 21138500

[44] Rao SS, Sadeghi P, Beaty J, Kavlock R Ambulatory 24-hour colonic manometry in slow-transit constipation. Am J Gastroenterol 99: 2405–2416, 2004. doi:10.1111/j.1572-0241.2004.40453.x. 15571589

[45] Rao SS, Sadeghi P, Beaty J, Kavlock R, Ackerson K Ambulatory 24-h colonic manometry in healthy humans. Am J Physiol Gastrointest Liver Physiol 280: G629–G639, 2001. doi:10.1152/ajpgi.2001.280.4.G629. 11254489

[46] Ritchie JA The gastrocolic response to food. Digestion 1: 15–21, 1968. doi:10.1159/000196827. 5671964

[47] Rodriguez L, Sood M, Di Lorenzo C, Saps M An ANMS-NASPGHAN consensus document on anorectal and colonic manometry in children. Neurogastroenterol Motil 29: e12944, 2017. doi:10.1111/nmo.12944. 27723185

[48] Rodriguez L, Siddiqui A, Nurko S Internal anal sphincter relaxation associated with bisacodyl-induced colonic high amplitude propagating contractions in children with constipation: a colo-anal reflex? Neurogastroenterol Motil 24: 1023-e545, 2012. doi:10.1111/j.1365-2982.2012.01965.x. 22757618 PMC3465462

[49] Sintusek P, Rybak A, Mutalib M, Thapar N, Borrelli O, Lindley KJ Preservation of the colo-anal reflex in colonic transection and post-operative Hirschsprung’s disease: Potential extrinsic neural pathway. Neurogastroenterol Motil 31: e13472, 2019. 10.1111/nmo.13472. 30288858 10.1111/nmo.13472

[50] Snape WJ Jr, Matarazzo SA, Cohen S Abnormal gastrocolonic response in patients with ulcerative colitis. Gut 21: 392–396, 1980. doi:10.1136/gut.21.5.392. 7429302 PMC1419080

[51] Snape WJ Jr, Wright SH, Battle WM, Cohen S The gastrocolic response: evidence for a neural mechanism. Gastroenterology 77: 1235–1240, 1979. doi:10.1016/0016-5085(79)90162-8. 583043

[52] Tambucci R, Quitadamo P, Thapar N, Zenzeri L, Caldaro T, Staiano A, Verrotti A, Borrelli O Diagnostic tests in pediatric constipation. J Pediatr Gastroenterol Nutr 66: e89–e98, 2018. doi:10.1097/MPG.0000000000001874. 29287015

[53] Taniyama K, Makimoto N, Furuichi A, Sakurai-Yamashita Y, Nagase Y, Kaibara M, Kanematsu T Functions of peripheral 5-hydroxytryptamine receptors, especially 5-hydroxytryptamine4 receptor, in gastrointestinal motility. J Gastroenterol 35: 575–582, 2000. doi:10.1007/s005350070056. 10955595

[54] Valentino RJ, Miselis RR, Pavcovich LA Pontine regulation of pelvic viscera: pharmacological target for pelvic visceral dysfunctions. Trends Pharmacol Sci 20: 253–260, 1999. doi:10.1016/S0165-6147(99)01332-2. 10366869

[55] van Lelyveld N, Ter Linde J, Schipper M, Samsom M Serotonergic signalling in the stomach and duodenum of patients with gastroparesis. Neurogastroenterol Motil 20: 448–455, 2008. doi:10.1111/j.1365-2982.2007.01068.x. 18208480

[56] Wang FB, Powley TL Topographic inventories of vagal afferents in gastrointestinal muscle. J Comp Neurol 421: 302–324, 2000. doi:10.1002/(SICI)1096-9861(20000605)421:3<302::AID-CNE2>3.0.CO;2-N. 10813789

[57] Wang YT, Yazaki E, Sifrim D High-resolution manometry: esophageal disorders not addressed by the “Chicago Classification”. J Neurogastroenterol Motil 18: 365–372, 2012. doi:10.5056/jnm.2012.18.4.365. 23105996 PMC3479249

[58] Wattchow D, Brookes S, Murphy E, Carbone S, de Fontgalland D, Costa M Regional variation in the neurochemical coding of the myenteric plexus of the human colon and changes in patients with slow transit constipation. Neurogastroenterol Motil 20: 1298–1305, 2008. doi:10.1111/j.1365-2982.2008.01165.x. 18662329

